# The Usefulness of Microalgae Compounds for Preventing Biofilm Infections

**DOI:** 10.3390/antibiotics9010009

**Published:** 2019-12-24

**Authors:** Yuly López, Sara M. Soto

**Affiliations:** Department, ISGlobal, Hospital Clínic-Universitat de Barcelona, 08036 Barcelona, Spain; yuly.lopez@isglobal.org

**Keywords:** microalgae, biofilm, antibiofilm activity, infection, quorum sensing

## Abstract

Biofilms play an important role in infectious diseases. It has been estimated that most medical infections are due to bacterial biofilms, and about 60–70% of nosocomial infections are also caused by the formation of a biofilm. Historically, microalgae are an important source of bioactive compounds, having novel structures and potential biological functions that make them attractive for different industries such as food, animal feed, aquaculture, cosmetics, and pharmaceutical. Several studies have described compounds produced by microalgae and cyanobacteria species with antimicrobial activity. However, studies on the antibiofilm activity of extracts and/or molecules produced by these microorganisms are scarce. Quorum-sensing inhibitor and anti-adherent agents have, among others, been isolated from microalgae and cyanobacteria species. The use of tools such as nanotechnology increase their power of action and can be used for preventing and treating biofilm-related infections.

## 1. Biofilms and Their Role in Infectious Diseases

**Figure 1:**
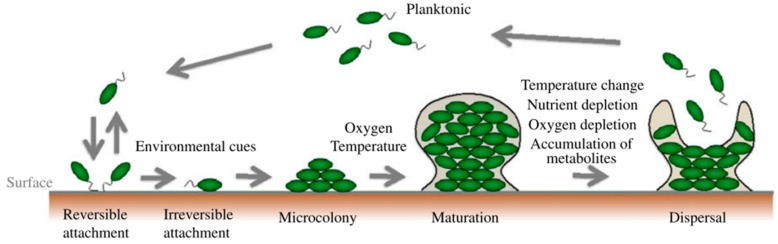
Biofilm formation and selected environmental factors that affect each stage [2].

Biofilms are defined as microbial communities of surface-attached cells embedded in a self-produced extracellular matrix and play an important role in infectious diseases. Biofilm formation is developed in three main stages: (i) attachment―the cells arrive to the surface and adhere to this surface; (ii) growth and maturation―the cells begin to produce the exopolysaccharide that constitutes the matrix and mature from microcolonies to multilayered cell clusters; (iii) detachment―the cells take on a planktonic state and can thereby form biofilm in other settings [1]. (Figure 1)

Bacteria inside a biofilm can be up to 1000-fold more resistant to antibiotics and can persist after treatment with antibiotics. Several mechanisms of antimicrobial resistance have been described in a biofilm: (i) poor diffusion of antibiotics through the polysaccharide matrix of the biofilm; (ii) physiological changes due to a slow growth rate and response to low oxygen, nutrient deprivation or environmental stress; (iii) phenotypic changes of the cells forming the biofilm; (iv) quorum-sensing; (v) the expression of efflux pumps; (vi) interchange of resistance genes among bacteria inside the biofilm; and (vii) the presence of persister cells―small fractions of persistent bacteria that resist death when exposed to antimicrobials [2]. In addition, these structures also allow biofilms to avoid the action of the immune system and evade phagocytosis.

It has been estimated that most medical infections are due to bacterial biofilms, and about 60–70% of nosocomial infections are also caused by the formation of a biofilm [3,4,5]. Biofilms are ubiquitous and can be located in different parts of the body, associated or not with medical devices or prostheses, causing different types of infections. Indwelling devices in which biofilm infections can be found include: intravenous catheters, vascular prosthesis, prosthetic heart valves, urinary catheters, joint prostheses and orthopedic fixation devices, peritoneal dialysis catheters, and intrauterine devices. Infections not associated with foreign bodies include cystic fibrosis (CF), chronic obstructive pulmonary disease, chronic otitis media, chronic sinusitis and chronic (diabetes) wound infections [3,4,6,7].

## 2. What is Needed to Fight Biofilms

Two important things are needed to fight biofilms: a diagnostic tool that can be used in clinical settings, and new molecules active against biofilm formation and/or against a mature biofilm. 

There is currently no standardized technique that can be routinely applied to determine whether or not a pathogen forms biofilm. Thus, treatment is based on the susceptibility of planktonic bacteria. Therefore, a rapid diagnostic tool able to determine if the isolated microorganism has the capacity to form biofilm, together the development of adequate treatment guidelines for these cases, could be avoid treatment failures in biofilm-related infections. In vitro and in vivo experiments have shown that the minimum inhibitory concentration (MIC) and the minimum bactericidal concentration (MBC) for biofilm bacterial cells are usually much higher (approximately 1–1000 times) than those of planktonic bacterial cells [4]. Moreover, MIC and MBC values are not applicable in vivo for biofilm eradication due to renal and hepatic toxicities. Therefore, a rapid diagnostic tool able to determine if the isolated microorganism has the capacity to form biofilm, is needed in addition to the development of adequate treatment guidelines for these cases in order to avoid treatment failures in biofilm-related infections.

The diagnosis of biofilm-related infections is complex and should combine a global multidisciplinary perspective which considers clinical aspects and microbiological findings. Initially, routine microbiological examinations including biopsies, culture, Gram staining, and susceptibility tests are important when infection is associated with a foreign body. However, in many cases, these tests are not sensitive enough to detect biofilm. Today, molecular analysis such 16S ribosomal RNA polymerase chain reaction (PCR) (bacteria) or 18S and 28S rRNA PCR (fungi) together with sonication of foreign body are effective complementary procedures in laboratories, due to the high sensitivity of pathogen detection, especially when microscopy and culture studies are negative in patients with clinical suspicion of biofilm infection [5].

Foreign bodies such as prostheses, urinary/venous catheters and prosthetic heart valves provide an ideal surface for the formation of biofilm. Once biofilm is established, the use of antimicrobials and immune response are not sufficient to eradicate the infection. In these cases, it is advisable to remove and/or replace the medical device to reduce the bacterial density and allow combined antibiotic therapy to be effective. However, in some cases such as in patients with central intravascular catheters, device replacement can be problematic and the risk of surgical removal may be greater than the risk associated with ineffective antibiotic treatment. In these cases, a treatment called locking is recommended, which consists of instilling a solution of an anticoagulant such as heparin and antibiotic inside the catheter at a concentration between 100 and 1000 times higher than the MIC of the microorganism responsible for the catheter-associated infection for at least eight hours a day during days 10–14. The antibiotics most commonly used in these cases are gentamicin, levofloxacin, cotrimoxazole, minocycline, teicoplanin, and vancomycin [8,9].

## 3. Prevention or Treatment of Biofilm Infections

Treatment of biofilm-related infections is a subject that requires further study. Several approaches to biofilm treatment have been developed. For example, clarithromycin blocks the biofilm matrix in *Pseudomonas aeruginosa* [10]. Ciprofloxacin reduces the overall thickness of the biofilm [11]. On the other hand, combination therapy has been recommended for the treatment of biofilm-related infections. Macrolides are one of the first antibiotics selected due to their high in vitro and in vivo antibiofilm activity against Gram-negative bacteria by the inhibition of alginate (one of the components of the matrix) production [12,13]. For example, the combinations of clarithromycin plus erythromycin [13] and clarithromycin plus vancomycin have in vitro antibiofilm activity against *P. aeruginosa* [14]. However, in the case of Gram-positive bacteria, macrolides enhance biofilm formation by the overexpression of several genes related to biofilm formation such as *ica*A, *atl*E, *fru*A, *pyr*R, *sar*A, and *sig*B [15]. 

Unfortunately, antimicrobial therapy in infections associated with foreign bodies is not sufficient due to the appearance of multidrug-resistant bacteria, including Gram-negative bacilli such as *P. aeruginosa,* which is associated with mechanical ventilation-associated pneumonia, *Escherichia coli* isolated from urinary tract catheter infection, and methicillin-resistant *Staphylococcus aureus* (MRSA) associated with prosthetic joints infection and heart valves [5]. For this reason, the need for new therapies to deal with this great problem is imperative.

**Figure 2:**
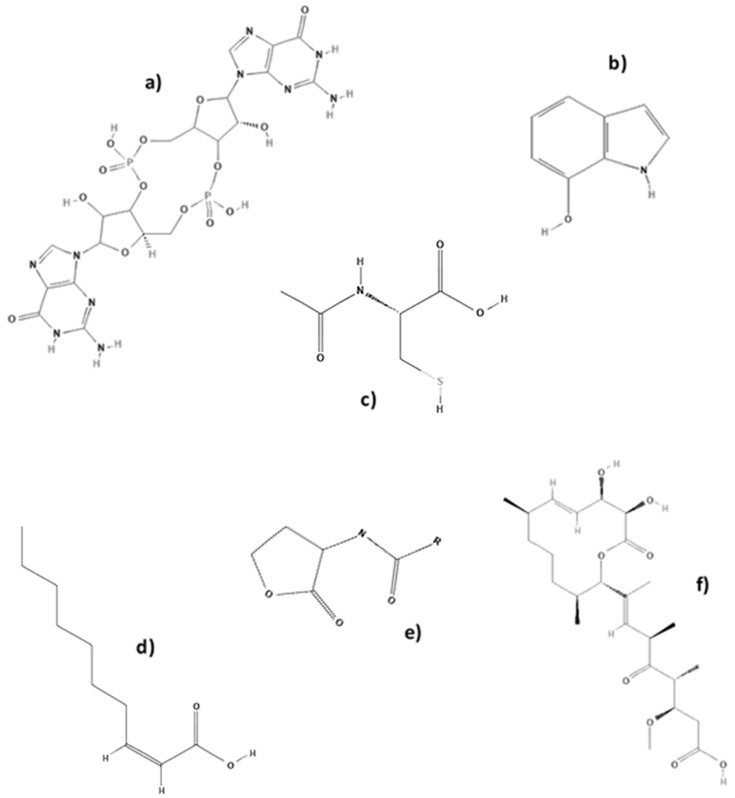
(**a**) Cyclic diguanylic acid (c-di-GMP), (**b**) 7-hydroxyindole, (**c**) N-acetylcysteine, (**d**) cis-2-decenoic acid, (**e**) acyl homoserine lactones (AHLs), and (**f**) carolacton.

According to these needs, several authors have focused their research on discovering “small molecules” with antibiofilm activity [3] (Table 1). Specifically, in 2013 Cheng and colleagues described the screening of more than 200,000 chemical compounds [16]. After screening 66,000 compounds and natural products in 2011, Sambanthamoorthy and col. [17] identified a small molecule called benzimidazole, which is able to inhibit biofilm formation by several Gram-negative and Gram-positive bacterial pathogens, including *P. aeruginosa* and *S. aureus*. Another important small molecule is the organic compound cis-2-decenoic acid isolate from *P. aeruginosa* that can inhibit biofilm development by a number of bacteria, including *E. coli*, *Klebsiella pneumoniae*, *Proteus mirabilis*, *Streptococcus pyogenes*, *Bacillus subtilis*, *S. aureus*, and *Candida albicans* [18]. (Figure 2)

Another strategy to combat biofilms is by avoiding the adherence of bacteria to surfaces or medical devices by bactericidal/bacteriostatic coating [29]. In this sense, catheters and prosthesis are coated with antibiotics or silver. Antibiotics such as vancomycin are commonly used. However, the use of antibiotics can lead to the selection of antibiotic resistant strains and even induce biofilm formation [30]. Silver has been used due to its broad-spectrum antimicrobial activity determined by the availability of the silver ion released to interact with the cell membranes of the microorganisms [31]. Nonetheless, while silver coating on medical devices prevents surface attachment of bacteria, it can have genotoxic and cytotoxic effects. On the other hand, anti-adhesion coatings such as silica colloids/silanexerogel, among others, can prevent the formation of bacterial biofilms. However, the in vivo efficacy of these coatings is still not clear due to the complexity of the interactions between coating surface with bacteria and host proteins [3,16].

Nevertheless, although there are also other experimental approaches to combat biofilms such as liposomes, bacterial interference, nanoparticles, hydrogels, iontophoresis, and bacteriophages, among others. New molecules with antibacterial and antibiofilm activity that can be used in clinical practice are needed. For this reason, it is important to explore new sources to find new molecules for combating biofilm-related infections.

Microalgae are one of the most promising sources of molecules/compounds with biological activity not only because of their high diversity of species and niches in which they live but also due to their high defence strategies against competitors and depredators.

## 4. Microalgae as a Source of Bioactive Compounds

In oceans, rivers, and lakes, microalgae represent an important group of microscopic and unicellular organisms that have the ability to perform photosynthesis, producing about half of the atmospheric oxygen [32].

The biodiversity of microalgae is enormous, and to date, only 50,000 species have been described among the 200,000–800,000 species estimated to exist. The current classification system of microalgae is based on different phenotypic characteristics such as morphology, photosynthetic pigments, cell wall composition and structure, sexual cycles and more recently, genomic studies by the comparison of 5S, 18S, and 28S ribosomal RNA sequences [33].

**Figure 3:**
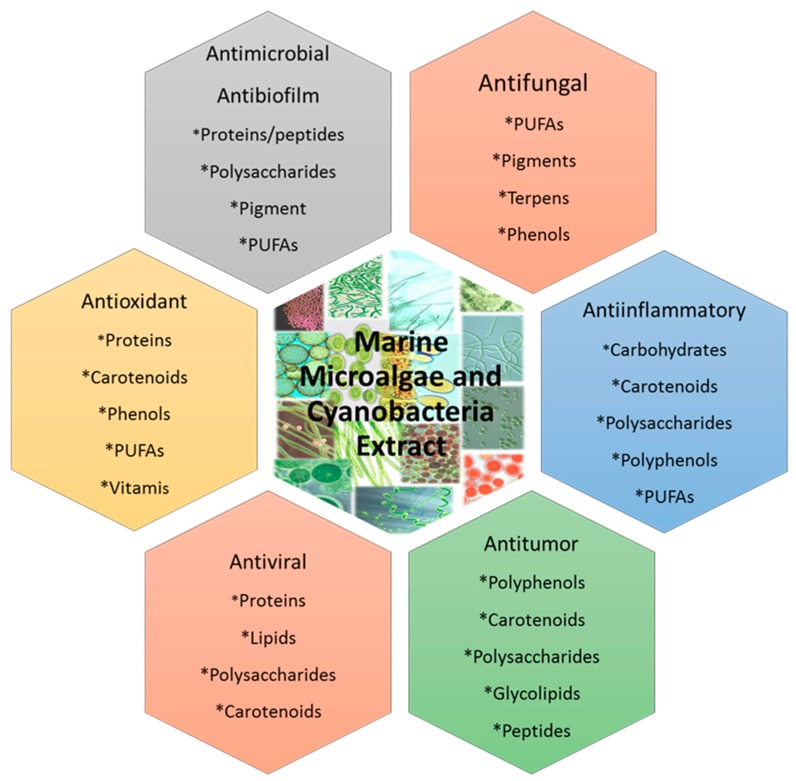
Properties of the biologically active compounds from marine microalgae and cyanobacteria.

Historically, microalgae are an important source of bioactive compounds with novel structures and potential biological functions that make them attractive for different food, animal feed, aquaculture, and cosmetics and pharmaceutical industries, among others [34,35,36] (Figure 3).

**Figure 4:**
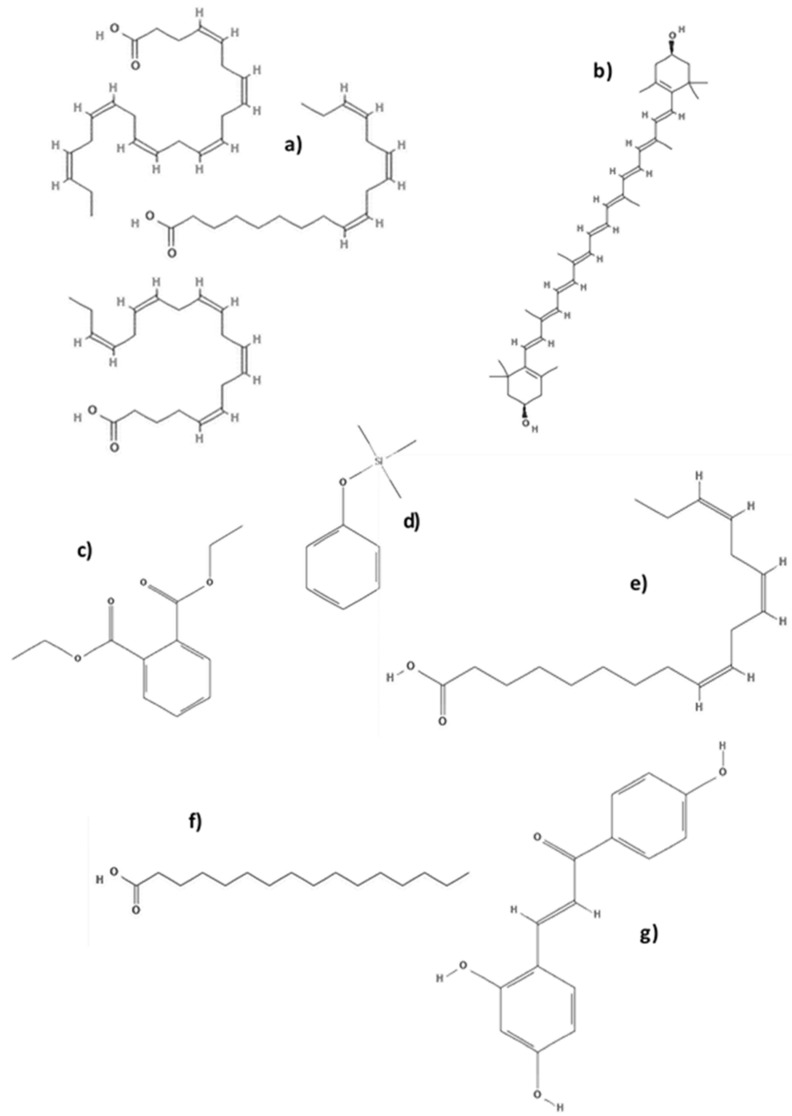
Molecules with antibiofilm activity isolated from microalgae (**a**) omega-3 fatty acids (PUFAs), (**b**) zeaxanthin, (**c**) diethyl phthalate, (**d**) trimethyl (4-tertbutyl phenoxy) silane, (**e**) linolenic acid, (**f**) palmitic acid, and (**g**) Glucosyltransferase-SI.

Currently, the global production of microalgae and cyanobacteria is predominately aimed at applications with high added value due to algal biomass containing pigments, proteins, toxins, polyunsaturated fatty acids (PUFAs), polysaccharides, vitamins, and minerals, all of which are of great interest in the preparation of natural products, as in food, cosmetics, and pharmaceutical industries [37,38,39]. (Table 2 and Figure 4)

Some of the main commercial microalgae products are PUFAs (**a**) used principally in the pharmaceutical and nutritional industries. Species such as *Chlorella* sp., *Spirulina* sp., *Dunaliella salina*, *Ochromonas* sp. and *Chlamydomonas reinhardtii* are a good source of these compounds [52].

Polysaccharides are another promising type of compounds due to their abundance among microalgae species. Several pharmaceutical, food and cosmetic industries use these compounds as moisturizing, thickening, stabilizer and emulsifier agents. [37,57]. Several types of polysaccharides such as homo-galactose, glucose, xylose, rhamnose, fucose and fructose showing antibacterial, antitumor, and antiviral properties can be extracted using “green” extraction techniques from the microalgae *Gymnodinium pudicum* and *Chorella vulgaris* [58,59,60].

The main microalgae species used in cosmetic products are *Arthrospira* sp, *D. salina,* and *Chlorella* sp., because they are an important source of pigments and natural colour enhancers. Among these isolated compounds, astaxanthin and carotenoid pigments are the most important for preventing oxidative stress caused by UV radiation [45].

Taking into account the ability of microalgae to combat pathogenic bacteria found throughout the different aquatic systems, they are considered one of the most important sources of antimicrobial molecules, including proteins, vitamins, fatty acids, pigments, etc [61]. In addition, microalgae coexist with other aquatic organisms creating competitive relationships that provoke them to synthetize or secrete several substances and/or molecules in order to inhibit their competitors [62]. It has been reported that some of these products have antimicrobial activity [62]. Among the compounds presenting antimicrobial activity discovered from marine organisms we can find alkaloids, terpenoids, lipids, peptides, halogenated compounds, polyketides, isocumarins, and nucleosides [63,64]. However, studies about antibiofilm activity of substances produced by microalgae are scarce.

Due to the global problem of antimicrobial resistance and the importance of bacterial forming biofilm as the cause of the most infections, there is an urgent need for new bioactive compounds with antibiofilm activity. 

## 5. Antibiofilm Activity of Compounds Isolated from Microalgae.

Several studies have described compounds produced by microalgae and cyanobacteria species with antimicrobial activity. However, studies on the antibiofilm activity of extracts and/or molecules produced by these microorganisms are scarce.

An important phase in the extraction methods of active compounds from microalgae is the choice of solvent. Crude microalgae extracts are a heterogeneous mixture of polar and non-polar compounds. The selection of an efficient method of extraction is important for performing successive assays. The solvents commonly used in this practice are hexane, chloroform, and petroleum ether (non-polar solvents), and ethyl acetate, methanol, acetone, and dichloromethane (polar solvents). A wide range of biological samples can be extracted in order to collect the greatest range of polar and non-polar compounds. For example, hexane can extract polar compounds, such as triacylglycerides (TAG), while polar solvents such as methanol and ethyl acetate can extract a wide variety of biological compounds (polar and non-polar metabolites) such as fatty acids (FA) [65].

Among the studies available in the literature, the study by Lauritano and col. should be highlighted [66]. The objective of their research was to find compounds with different biological activities, including antibacterial and antibiofilm activities from crude extracts of 32 microalgal species, including diatoms, dinoflagellates, and flagellates. Among these species, while the *Leptocylindrus* genus did not show cytotoxicity in previous antibacterial tests, it has strong antibiofilm activity, inhibiting 90% of *S. epidermidis* when the microalgae are grown under stress conditions caused by nitrogen starvation. Therefore, this environmental stress could be the reason for the greater production of molecules with antagonistic activity against clinically important bacteria.

Recently, in the NoMorFilm project in 2019, Cepas and col. [67], investigated the antibiofilm activity of 675 extracts from 225 microalgae and cyanobacteria species, including the phylum Cercozoa, Charophyta, Chlorophyta, Cryptophyta, Cyanobacteria, Euglenophyta, Glaucophyta, Haptophyta, Miozoa, Ochrophyta, Rhodophyta, and two unknown phyla, against clinically important bacterial and fungal pathogens.

**Figure 5:**
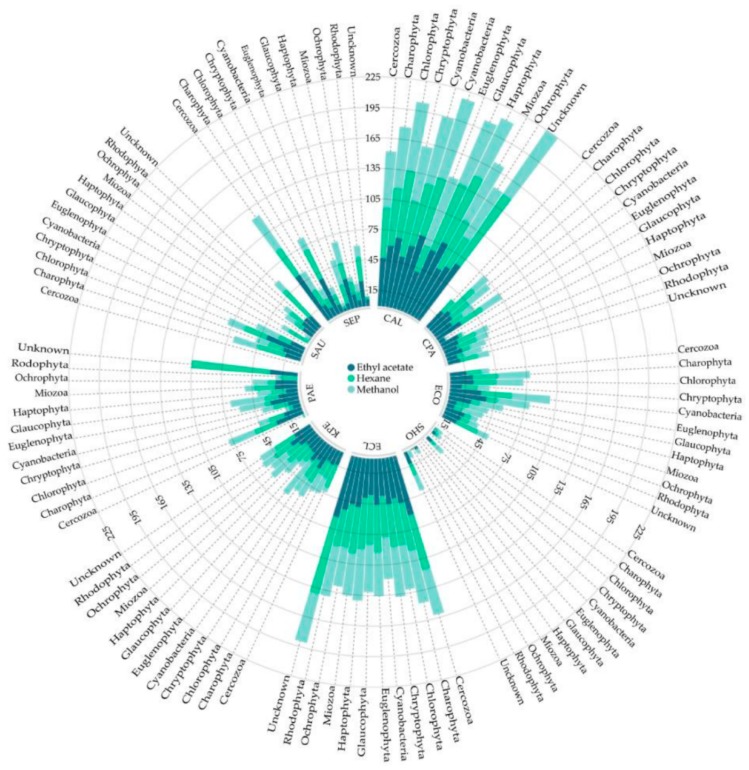
Circular dot plot representing the biofilm inhibition ratio (%) of each bacterium in relation to the solvent employed (ethyl acetate, hexane, and methanol), according to the microalgae and cyanobacteria phylum. CAL: *C. albicans;* CPA: *C. parapsilopsis*; ECO: *E. coli*; SHO: *S. hominis*; ECL: *E. cloacae*; KPE: *K. pneumoniae*; PAE: *P. aeruginosa*; SAU: *S. aureus*; SEP: *S. epidermidis*. [67].

In this work, the extraction method consisted of three different solvents (hexane [non-polar], ethyl acetate [polar], and methanol [polar]) for extracting a wide range of biological samples. The highest inhibition ratios of extracts from all the solvents were found against *C. albicans* and *E. cloacae*. Interestingly, *C. albicans* showed high inhibition rates above 50% in all the samples, with the exception of Glaucophyta and Miozoa methanol extracts (28.2% and 12.55%, respectively) and Rhodophyta hexane extracts (34.77%). In the case of *E. cloacae*, the biofilm inhibition ratios were 35%, and only the methanol extract from the Miozoa phylum showed activity below 35% (9.38%). (Figure 5)

On the other hand, Chlorophyta and Charophyta extracts showed higher minimal biofilm inhibitory concentration (MBIC) values than the other phyla. Curiously, the Chlorophyta phylum is known for its ability to synthesize a variety of bioactive compounds such as lipids and derivative polyunsaturated fatty acids (PUFAs). Specifically, two PUFAs, docosahexaenoic acid (DHA) and eicosapentaenoic acid (EPA), have demonstrated to exhibit antibacterial and antibiofilm properties [68,69]. However, in this study, their presence was not evident.

An important focus in the search for eco-friendly bioactives is to investigate molecules that have the cell-cell communication system responsible for population density as a target. It is well-known that bacteria present inside biofilm communicate with each other through signalling molecules called autoinducers in response to population density. This process is known as quorum sensing (QS) [3,70]. Gram-negative and Gram-positive bacteria use acylated homoserine lactones and oligopeptides as autoinducers which induce the expression of QS genes [71,72].

In this sense, the freshwater green microalgae *Chlorella vulgaris* produces a high variety of bioactive compounds with important nutritional qualities and interesting therapeutic properties. Studies using *Chlorella* mainly demonstrated its antitumoural effects, cancer chemoprevention properties, anti-inflammatory activity, antioxidant activity, immune system activation, antimicrobial, and antibiofilm activity [73].

Taking into account this background, Varsha Gayatri and col, [50] from the Valliammal College for Women in India, investigated the antibiofilm effect of *Chlorella vulgaris* ethanolic extracts on biofilm development and QS inhibition. The results of this investigation, showed that the inhibition of biofilm formation by these ethanolic extracts was around 85% in *P. aeruginosa* at a concentration of 2 mg/mL, 80% at 1 mg/mL, 75% at 0.5 mg/mL, 70% at 0.25 mg/mL, 65% at 0.125 mg/mL, and 60% at 0.0625 mg/mL. In addition, scanning electron microscope (SEM) showed a significant reduction in biofilm formation at a concentration of 1 mg/mL, demonstrating this inhibitory activity.

Interestingly, the ethanolic extracts were able to dramatically reduce the biofilm without inhibiting growth, which was associated with an interruption of “sensing” but not with a reduction of “quorum”. Analysis by gas-chromatography and mass spectro-photometry (GS–MS) revealed that diethyl phthalate at Rt 15.563 (39.28%) and trimethyl (4-tertbutyl phenoxy) silane at Rt 31.944 (25.63%), were the major compounds in the ethanolic extract of *C. vulgaris*, and could play an important role in antibiofilm strategies against multidrug-resistant *P. aeruginosa*. Similar results were found in 2015 by Lewis Oscar. and col. [55] who observed that the *Spirulina* extract has strong inhibitory biofilm formation activity but does not exhibit antibacterial activity.

In addition, researchers from India, [74] determined the antibiofilm activity of methanolic extracts of *C. vulgaris* against *P. aeruginosa* and *S. aureus*. Treatments of 1.0, 0.5, and 0.25 mg/mL of this extract showed significant reductions of biofilm formation in *P. aeruginosa* (82.5%, 56.5%, and 46.5%, respectively) and in *S. aureus* (88.0%, 58.5%, and 48.0%, respectively).

Another important area of research is oral health. Dental caries are the result of activities of bacterial strains [75]. The main microorganisms associated with tooth caries are *Streptococcus mutans* and *Lactobacillus* sp. The erosion of tooth enamel by bacteria is due to the generation of acids as a result of the metabolism of sugar fermentation, and the ability that these microorganisms can withstand acidic conditions in dental plaque [76]. Furthermore, the development of biofilm formation associated with *S. mutans* could explain the appearance of a variety of diseases in the oral cavity. In this sense, the ethanolic extracts of *C. vulgaris* and *D. salina* could contribute to the inhibition of biofilm formation.

The 2019 disc diffusion results of Jafari [77] showed the antimicrobial activity of *C. vulgaris* extract against *S. mutants* at concentrations of 2.5 mg/disc, producing a large inhibition zone with a diameter of 13.5 ± 0.92 mm. In the case of a *D. salina* extract, a concentration of 6 mg/disk showed an inhibition diameter of 18.5 ± 0.97 mm. With respect to antibiofilm activity, a *C. vulgaris* extract showed antibiofilm activity at a concentration of 4 mg/mL, while for the *D. salina* extract 2 mg/mL was necessary to inhibit biofilm formation. Although *C. vulgaris* and *D. salina* have been investigated by many authors broadly for their biological activity [78], this was the first study to demonstrate the effects of these microalgae against viability and biofilm formation of *S. mutans*. According to the analysis of the extracts, the authors suggested that flavonoids, tannins, and terpenoids from *C. vulgaris* extract and 3,3,5-trimethylheptane, n-hexadecane together with PUFAs and β-ionone and neophytadiene (related to carotene metabolism) from *D. salina* extract might be responsible for antimicrobial properties. On the other hand, the suppressive activity of glucosyltransferases (GTF) from *C. vulgaris* and *D. salina* might be attributed to the antibiofilm activity.

Currently, nanotechnology is becoming increasingly used in the exploration of different fields such as chemistry, biology, physics, materials science, and engineering. The production of nanodevices has been useful in various applications and could be a very attractive tool for the treatment of certain infectious diseases. In the general context, the unique properties possessed by nano-materials are a result of their small size (range from 0.1 to 100 nm) and the large surface to volume ratio and definite quantum confinement. [79,80].

Production of nanoparticles can be achieved through traditional physical, biological and chemical methods, with the last method commonly involving the use of toxic chemicals. However, the main challenge of nanotechnology today is the biosynthetic production of eco-friendly nanoparticles which do not represent a threat to the environment or humans as a result of their administration [81,82].

Silver nanoparticles (AgNPs) have shown outstanding antimicrobial properties unlike standard antimicrobial agents. Low doses of silver nanoparticles are needed for the treatment of infectious diseases. Their high antimicrobial activity against Gram-negative and Gram-positive strains is mainly due to the release of silver ions, which act as potential destroyers of microbial cells, generating an alteration of cell permeability followed by cell lysis and apoptosis [83,84].

Along the “eco-friendly”line, in 2018 Adebayo-Tayo and colleagues [82] worked on the biosynthesis of silver nanoparticles containing methanol extract of the *Oscillatoria* sp green algae. The results of this research showed the biosynthesized OsSNPs to have strong antibacterial activity against seven clinical pathogenic bacteria (*S. aureus* ATCC29213, *E. coli* ATCC 11775, *E. coli* ATCC 35218, *P. aeruginosa* ATCC27853, *Citrobacte*r sp., *S. typhi* ATCC 14028, and *Bacillus cereus*) at 24, 48, and 72 h. The inhibition zone ranged between 6 mm for *B. cereus* to 20 mm for *E. coli*. In addition, studies with *Oscillatoria* sp. extract showed antibacterial activity against other pathogenic bacteria with an inhibition range from 13–24 mm, with the highest activity being against *P. aeruginosa*. In addition, these OsSNPs showed strong antibiofilm activity against all the pathogens used in the study. The highest biofilm inhibition was observed against *P. aeruginosa* ATCC 27853 while the lowest inhibition was against *Citrobacter* sp. 

Another study in the “eco-friendly” line was carried out by Vijayan and col. in 2014, [85]. They observed that after 24 h of incubation, the AgNPs associated with aqueous extract of *Turbinaria conoides* (macroalgae) inhibited the adherence and biofilm formation of *Salmonella* sp., *E. coli*, *Serratialique faciens*, and *Aeromonas hydrophila* at very low concentrations.

The use of nanoparticles in the discovery of new compounds with antibiofilm activity has been reported previously by other authors. In 2013, Martinez-Gutierrez and colleagues [86] evaluated the antibiofilm activity of AgNPs against biofilms generated on membranes under static and high fluid conditions. The bacterial strains evaluated were *P. aeruginosa*, *A. baumannii*, MRSA (as colonizer of medical devices), *S. mutans* and *C. albicans* (as cariogenic and periodontopathogenic pathogens). The results of this work showed an important reduction of 4-log in the number of colony-forming units?? of biofilm in *P. aeruginosa* exposed to 500 mg/mL of AgNPs and a 6-log reduction when the concentration of the AgNPs was increased to 1000 mg/mL. In addition, important results were found with *A. baumannii*, observing a reduction of 3.5–4-log when cells were exposed to AgNPs concentrations between 250 and 1000 mg/mL. A reduction of only 1-log was observed in the remaining microbial strains.

## 6. Conclusions

Many different compounds with important antibacterial and antibiofilm properties have been isolated from microalgae. However, studies on the mechanisms of action and toxicity of these compounds are needed. An interesting line of research with promising results is the study of metabolites synthesized by natural organisms such as marine microalgae. These molecules could increase their power of action through the use of tools such as nanotechnology and could be useful for preventing and treating biofilm-related infections.

## 7. Future Aspects

Marine organisms, including microalgae, are one of the most important sources of biomolecules with antimicrobial and anticancer activities, among others that could be useful for the treatment and prevention of many human diseases. Nonetheless, one of the problems with these molecules is the small amount in which they are present in the extracts. For this reason, it is necessary to determine different ways to increase the production of these molecules. Taking this into account, new molecular biology techniques such as CRISP/Cas9 and genomic studies could help to obtain larger amounts of molecules without the need for a high amount of reagents and time, thereby decreasing the costs associated with this production.

To achieve this, collaboration among research groups from different disciplines (chemistry, biology, microbiology, etc.) is needed to identify these molecules and new processes to improve their production without effects against their natural sources. In addition, the pharmaceutical industry should become more involved in supporting the development of the new molecules to reach the market.

## Figures and Tables

**Table 1:** Small molecules with antibiofilm activity.

Molecule	Mechanism	Effect	Ref.
Anti-virulence compounds	Inhibition of gene expression of virulence factors	Inhibition of biofilm formation by *S. aureus*	[19]
Anti-biofilm compounds	Unknown	Inhibition of biofilm formation by *S. epidermidis*	[20]
Acyl Homoserine Lactones (AHLs)	Autoinducers-QS	Inhibition of biofilm formation by *P. aeruginosa*	[21]
Autoinducing peptides (AIPs)	Signaling molecules	Inhibition of biofilm formation by *S. aureus*	[22]
ABC-1	Inhibition of c-di-GMP-inducible transcription	Inhibition of biofilm formation by multiple Gram-negative and Gram-positive bacterial pathogens	[17]
Indole and derivatives	Oxidized indole metabolites	Inhibition of biofilm formation by *E. coli, P. aeruginosa,* Staphylococcal species	[23]
Carolacton	Affect the expression of two component signal transduction systems	Inhibition of biofilm formation by *S. mutans*	[24]
Chelators	Interference with metal ion’s function in biofilm formation	Inhibition of biofilm formation by *S. aureus*	[25]
Aryl rhodanines	Unknown	Inhibition of biofilm formation by *S. aureus* and *S. epidermidis*	[26]
Cis-2-decenoic acid	Unknown	Dispersion of biofilms by *E. coli*, *K. pneumoniae*, *P. mirabilis*, *S. pyogenes*, *B. subtilis*, *S. aureus*, and *C. albicans*	[18]
D-amino acids	Unknown	Inhibition of biofilm formation by *S. aureus* and *P. aeruginosa*	[27]
N-acetylcysteine	Interference with exopolysaccharide formation in biofilms	Inhibition of biofilm formation by *S. epidermidis*	[28]

**Table 2:** Principal bioactive compounds extracted from microalgae.

Microalgae	Bioactive Compounds	Use	Ref.
*Arthrospira platensis*	Methanolic extracts of exopolysaccharides	Antioxidant	[40]
*Botryococcus braunii*	Linear alkadienes (C25, C27, C29, and C31), triene (C29)	Phycoremediation	[41]
*Chlorella sp.*	Carotenoids, sulfated polysaccharides, sterols, PUFAs (n-3) (a) fatty acids, chlorophyll	Moisturizing and thickener agent, dentifrices and deodorants, antimicrobial, antibiofilm	[42,43,44]
*Chlorella ellipsoidea*	Zeaxanthin (b), violaxanthin	Health and cosmetic as UV protection, antioxidant and antibiofilm	[45]
*Chlorella minutissima*	Eicosapentaenoic acid (EPA)	Food supplements	[46]
*Chlorella protothecoides*	Lutein, zeaxanthin, canthaxanthin	Health and cosmetic as UV protection, antioxidant	[47,48]
*Chlorella pyrenoidosa*	Lutein, sulfated polysaccharide	Health and cosmetic as UV protection, antioxidant	[47,49]
*Chlorella vulgaris*	Canthaxanthin, astaxanthin, peptide, oleic acid, Diethyl phthalate (c), trimethyl (4-tertbutyl phenoxy) silane (d), *chlorella vulgaris* extracts	Antioxidant, antimicrobial, antibiofilm, anti-ageing	[49,50,51]
*Chlorella zofingiensis*	Astaxanthin	Health and cosmetic as UV protection, antioxidant	[51]
*Dunaliella salina*	Trans-betacarotene, cis-betacarotene, β-carotene, oleic acid, linolenic (e) acid, palmitic acid (f), β-Cryptoxanthin and glucosyltransferases (GTF) (g)	Health and cosmetic as UV protection. Anti-inflammator, antibacterial and antibiofilm.	[43,52]
*Dunaliella* sp	Diacylglycerols	Acylation stimulating protein	[53]
*Haematococcus pluvialis*	Astaxanthin, lutein, zeaxanthin, canthaxanthin, lutein, β-carotene, oleic acid	Health and cosmetic as UV protection, antioxidant	[54]
*Oscillatoria* sp	*Oscillatoria* sp. extract	Antioxidant, antimicrobial, antibiofilm	[51]
*Spirulina* sp	Polysaccharides	Food and in cosmetics	[37]
*Spirulina platensis*	Phycocyanin, C-phycocyanin, phenolic acids, tocopherols (vitamin E), neophytadiene, phytol, PUFAs (n-3) fatty acids, oleic acid, linolenic acid, palmitoleic acid	Food, health and cosmetics, antimicrobial, antibiofilm	[55]
*Spirulina fusiformis*	Diacylglycerols	Acylation stimulating protein	[51]
*Nostoclinckia/Nostocspongiaeforme*	Borophycin, cryptophycin	Anti-tumor compounds	[56]
